# Identifying Associations between DCE-MRI Radiomic Features and Expression Heterogeneity of Hallmark Pathways in Breast Cancer: A Multi-Center Radiogenomic Study

**DOI:** 10.3390/genes14010028

**Published:** 2022-12-22

**Authors:** Wenlong Ming, Yanhui Zhu, Fuyu Li, Yunfei Bai, Wanjun Gu, Yun Liu, Xiao Sun, Xiaoan Liu, Hongde Liu

**Affiliations:** 1 State Key Laboratory of Bioelectronics, School of Biological Science and Medical Engineering, Southeast University, Nanjing 210096, China; 2 Division of Medical Image Computing, German Cancer Research Center (DKFZ), Im Neuenheimer Feld 280, 69120 Heidelberg, Germany; 3 Department of Breast Surgery, The First Affiliated Hospital of Nanjing Medical University, 300 Guangzhou Road, Nanjing 210029, China; 4 Collaborative Innovation Center of Jiangsu Province of Cancer Prevention and Treatment of Chinese Medicine, School of Artificial Intelligence and Information Technology, Nanjing University of Chinese Medicine, Nanjing 210023, China; 5 Department of Information, The First Affiliated Hospital of Nanjing Medical University, 300 Guangzhou Road, Nanjing 210029, China

**Keywords:** radiogenomics, association analysis, breast cancer, DCE-MRI, pathway, radiomics, machine learning

## Abstract

Background: To investigate the relationship between dynamic contrast-enhanced magnetic resonance imaging (DCE-MRI) radiomic features and the expression activity of hallmark pathways and to develop prediction models of pathway-level heterogeneity for breast cancer (BC) patients. Methods: Two radiogenomic cohorts were analyzed (*n* = 246). Tumor regions were segmented semiautomatically, and 174 imaging features were extracted. Gene set enrichment analysis (GSEA) and gene set variation analysis (GSVA) were performed to identify significant imaging-pathway associations. Random forest regression was used to predict pathway enrichment scores. Five-fold cross-validation and grid search were used to determine the optimal preprocessing operation and hyperparameters. Results: We identified 43 pathways, and 101 radiomic features were significantly related in the discovery cohort (*p*-value < 0.05). The imaging features of the tumor shape and mid-to-late post-contrast stages showed more transcriptional connections. Ten pathways relevant to functions such as cell cycle showed a high correlation with imaging in both cohorts. The prediction model for the mTORC1 signaling pathway achieved the best performance with the mean absolute errors (MAEs) of 27.29 and 28.61% in internal and external test sets, respectively. Conclusions: The DCE-MRI features were associated with hallmark activities and may improve individualized medicine for BC by noninvasively predicting pathway-level heterogeneity.

## 1. Introduction

Breast cancer (BC) is currently the most frequent malignant tumor worldwide (11.7% of new tumors in 2020), with an estimated 2.3 million new cases, and the death rates of BC were 15.0 and 12.8 per 100,000 cases in developing and developed countries, respectively, indicating that BC is a serious health threat to women [1]. The high degree of heterogeneity poses a challenge to accurate diagnosis and the decision of treatment strategy in the clinical practices of BC [2,3]. Based on the expression profiling of 50 important genes, BC can be categorized into five major molecular subtypes, including luminal-A, luminal-B, HER2-enriched, basal-like, and normal-like, which is also known as the prediction analysis on microarray (PAM50) intrinsic molecular subtypes [4,5]. Different PAM50 subtypes of BC have distinct risks of recurrence, metastasis, and response to treatment [6,7]. Accurate and timely identification of the intrinsic molecular subtypes of patients is essential for the clinical management of BC. However, due to the high cost of RNA-sequencing, immunohistochemistry (IHC) testing is the commonly used alternative method in clinics to identify the molecular characteristics of BC by detecting the gene expression of four specific biomarkers, including the estrogen receptor (ER), progesterone receptor (PR), human epidermal growth factor receptor 2 (HER2), and Ki67 [8,9]. These findings on the transcriptomic heterogeneity of BC, including both the intrinsic expression pattern and status of the IHC biomarker, have greatly contributed to precision medicine for BC and improved overall patient outcomes [10,11]. Most of these studies were based on a single gene or a small number of genes and have been highly successful. The expression heterogeneity at the level of the pathway is also considered a very important feature of BC, but a lot of work is still needed in this area [12,13,14]. For example, the hallmark pathways are derived from various overlapping original gene sets, which can reduce noise and redundancy and provide a better delineated biological space to characterize the transcriptomics of disease [15]. Non-invasive access to the heterogeneity at the hallmark pathway level might contribute to personalized medicine for BC.

In the last decade, with the advancement of data science and artificial intelligence technologies, radiomics has been rapidly developed within the field of oncology research, including BC [16,17]. Identifying the reliable associations between quantitative medical imaging features and vital information about the disease, such as genetic mutations, molecular or clinical features, and treatment responses, as well as non-invasively predicting these cancer characteristics from imaging features are the core goals of radiomic research. As a subset of radiomics, radiogenomics focuses on analyzing the association of tumor genomic or transcriptomic characteristics with imaging features [18,19]. The association between transcriptomic characteristics and imaging features has been investigated in BC [20,21,22,23,24], for example, the distinct expression activity of cell cycle pathways was related to the imaging characteristics and clinical outcomes of BC patients [21]. However, the current studies have some shortcomings. First, most of the associations between the transcriptomic characteristics and imaging features in previous BC radiogenomics studies have been reported in a single dataset only, lacking the validation of an independent dataset. Second, few studies have analyzed the association between pathway heterogeneity and imaging features, especially for the representative hallmark gene sets. Finally, to our knowledge, no radiomic feature-based machine learning prediction models of pathway expression activity in individual BC patients have been built and reported.

**Figure 1:**
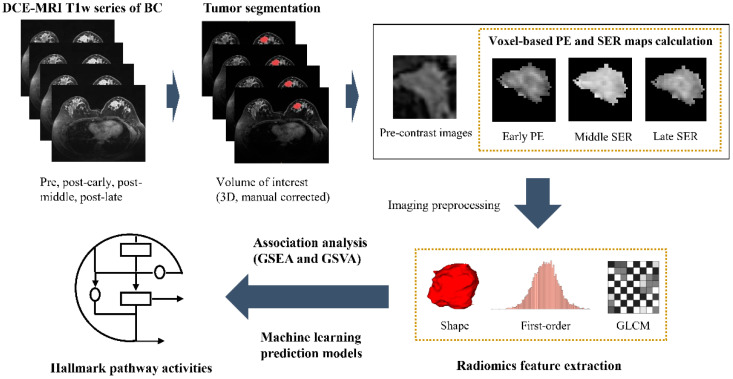
The workflow of this DCE-MRI- and RNA-Seq-data-based radiogenomics study.

In this work, to explore the associations between the imaging features of BC patients and the transcriptional heterogeneity of the hallmark pathways, two radiogenomics cohorts were collected from local and public databases. The workflow of this radiogenomic study is illustrated and detailed in Figure 1. The dynamic contrast-enhanced MRI (DCE-MRI) data and RNA-Seq data from the radiogenomics cohorts were analyzed. DCE-MRI is the most accurate modality for BC imaging now with the advantages of high resolution and temporal contiguity. Using contrast agents, DCE-MRI can amplify the differences between tumor tissue and normal tissue, which helps to reflect the imaging characteristics of tumors. The discovery cohort, including 174 patients, was used to identify the associations, and the multi-center validation cohort, including 72 patients, was used to validate. Then, the preference for significant transcriptomic–radiomic correlations was further analyzed and reported for the first time. Namely, different types of imaging features were associated with diverse pathway activities, and, correspondingly, hallmark pathways with various biological functions displayed distinct imaging correlations. Finally, we developed machine learning regression models for the enrichment scores (ESs) of some key hallmark pathways in BC based on the DCE-MRI radiomic features in an attempt to non-invasively predict pathway expression heterogeneity in individual patients. We hope this exploratory work can contribute to precision medicine in BC.

## 2. Materials and Methods

### 2.1. Subjects and Pathology Evaluation

This study was approved by the local institutional ethics committee. Written informed consent was obtained for each local patient because patients had been informed that their data, including specimens and images, might be used for possible research in the future. We employed two independent radiogenomic cohorts from multiple medical centers. The discovery cohort was collected from the local database between August 2016 and December 2018 and included 174 BC patients whose preoperative T1-weighted DCE-MRI and tumor specimens were both available. Data of DCE-MRI and RNA-seq of the external validation cohort were retrieved from the Cancer Imaging Archive (TCIA) and the Cancer Genome Atlas (TCGA) databases from four centers, including the Memorial Sloan Kettering Cancer Center, Mayo Clinic, University of Pittsburgh Medical Center, and Roswell Park Cancer Institute [25]. The detailed inclusion and exclusion criteria of both cohorts are displayed in Figure S1, and finally, we obtained and analyzed 174 and 72 patients in the discovery and validation cohorts, respectively.

ER, PR, HER2, and Ki67 were used to determine the clinical immunohistochemistry (IHC) subtypes for each patient in the discovery cohort. ER-positive, HER2-negative, high-PR expression (more than 20%), and low-Ki67 expression (less than 20%) subjects were defined as luminal-A. ER-positive, HER2-negative, low-PR, or high-Ki-67 expression subjects were defined as luminal-B. In addition, ER- and HER2-positive subjects were defined as luminal-B. ER, PR-negative, and HER2-positive patients were HER2-positive, and finally, all negative samples were triple-negative BC (TNBC).

### 2.2. DCE-MR Imaging

The DCE-MR images of the discovery cohort were scanned and acquired using Siemens TrioTim 3-Tesla (3T) scanner (Siemens Healthcare, Erlangen, Germany) in the axial position. Gadolinium-diethylenetriamine pentaacetic acid (Gd-DTPA) was used as the contrast agent, and patients were injected with the agent at an amount of 15 mL in a dose of 0.1 mmol/kg. The dynamic T1-weighted MRI sequences with fat suppression were acquired at six-timepoints, including one pre-contrast and five post-contrast (time interval was approximately 1 min). For most images from the discovery cohort, the echo time was 15.7 ms, the repetition time was 423 ms, the flip angle was 10 degrees, the field of view was 340 × 340 mm, the matrix size was 448 × 448 pixels, and the slice thickness was 0.9 mm. For the multi-center validation cohort, using the T1-weighted 3D spoiled gradient-echo sequence, GE scanners (GE Healthcare, Chicago, IL, USA) on a 1.5 Tesla magnet strength scanned and generated the images. The in-plane resolution of validation images ranged from 0.53 to 0.86 mm, spacing between slices ranged from 2 to 3 mm, the flip angle was 10 degrees, and the acquisition matrix was 256 × 192.

### 2.3. Segmentation of Tumor Regions

Before extracting the quantitative radiomic features, tumor segmentation, and image pre-processing were conducted. Firstly, based on the differences in kinetic characteristics between tumor tissue and normal tissue during dynamic contrast enhancement, we segmented and obtained the rough volume of interest (VOI) of the tumor mask by setting a threshold of intensity value on the registered and subtracted images of the first enhanced MRI sequences in the open-source software 3D Slicer (version 4.8.1, Cambridge, MA, USA). Secondly, the tumor masks were corrected manually and finally confirmed by two radiologists blinded to the clinical information, one with 10 years and another with 3 years of breast imaging experience.

### 2.4. Calculation of Percentage Enhancement and Signal Enhancement Ratio Maps

By taking advantage of the DCE-MRI technique, we defined and calculated the voxel-based percentage enhancement (PE) and signal enhancement ratio (SER) maps based on the signal intensity of each voxel for each patient in this work [23,26,27]. Firstly, the N4 bias correction algorithm was used to reduce the possible heterogeneity bias of 3T MR images [28]. Then, with the purpose of extracting the dynamic radiomic features of signal intensity in the tumor region after contrast agent injection, MRI sequences at four timepoints, including pre-enhanced (scan before contrast injection), early, middle, and late post-enhanced images (scans at approximately 1, 3, and 5 min after contrast injection), were selected and used to calculate the intensity ratio maps. The early PE map for each patient was acquired using Equation (1):

(1) $$PE_{early}=100\times \frac{I_{early}-I_{pre}}{I_{pre}}$$

where $I_{early}$ was the signal intensity of each voxel in early enhancement, and $I_{pre}$ was the voxel initial intensity before contrast. The SER maps of both middle and late enhancement were calculated using Equation (2):

(2) $$SER_{map}=\frac{I_{early\;}-I_{pre}}{I_{map}-I_{pre}}$$

where $I_{map}$ here is the voxel-enhanced signal intensity in middle or late images. After that, we performed the subsequent radiomic feature extraction using pre-contrast MR images and early PE, middle, and late SER maps. The map calculation was conducted in Python 3.5.2 (Virginia, USA).

### 2.5. DCE-MRI Radiomic Feature Extraction

An open-source Python package, pyradiomics (version 2.2.0), was used to perform image normalization and feature calculation [29]. Image normalization included remapping the histogram to fit within μ ± 3σ (μ means gray level within the volume of segmentation; σ means gray-level standard deviation) and resampling images to an isotropic voxel resolution of 1 mm using the B-spline method. Finally, according to the image biomarker standardization initiative (IBSI), 174 quantitative radiomic features, including 14 tumor-shape features, 72 histogram features, and 88 gray-level co-occurrence matrix (GLCM) features, were calculated for each patient. All procedures of image pre-processing and feature extraction were completed in Python 3.5.2 (Virginia, USA).

### 2.6. RNA-Sequencing and Bioinformatics Analysis

Tumor tissue of 199 BC patients was collected in the local database. The total RNA was obtained using VAHTS Total RNA-seq (H/M/R) Library Prep Kit for Illumina with the manufacturer protocol and stored in liquid nitrogen at −80 °C. Ovation human FFPE RNA-seq library systems were used to construct RNA-seq libraries, and RNA was sequenced on an Illumina HiSeq X Ten platform using paired-end 150 bp runs. We used Trimmomatic to control the sequencing quality [30]. Then, the RNA-seq reads were aligned to human genome 19 using STAR29 and quantified using HTSeq-Count [31,32]. Finally, expression values of 57,773 transcripts were quantified in the forms of both counts and FPKM (fragments per kilobase of exon per million reads mapped) for each patient in the discovery cohort.

Both the qualitative and quantitative associations between 174 imaging features and the 50 hallmark pathways (defined in http://www.gsea-msigdb.org/gsea/msigdb/human/genesets.jsp?collection=H, accessed on 20 January 2020) were investigated in this work. To obtain the qualitative associations, patients were classified into high and low groups for each imaging feature using the median as the cutoff. The gene differential expression analysis between the high and low groups was then conducted using DESeq2 [33]. After that, the gene set enrichment analysis (GSEA) was performed to identify the hallmark gene sets that were significantly enriched in the high- or low-imaging feature value groups by determining the positions of the genes of a hallmark pathway in the ranked all gene list based on differential expression analysis. The P values of hallmark–imaging correlation pairs were corrected using false discovery rate (FDR), and an FDR-corrected P value less than 0.05 could finally be retained [34]. The quantitative associations were calculated using the gene set variation analysis (GSVA) and Pearson correlation coefficient. GSVA was performed using the R package GSVA based on the RNA-seq data to obtain the enrichment scores (ESs) of pathways in individuals [35], and Pearson correlation coefficient was used to calculate the relationships between radiomic features and ESs.

### 2.7. Feature Pre-Processing and Machine Learning Modeling

To non-invasively predict the enrichment scores (ESs) of GSVA of hallmark pathways from radiomic features, the random forest (RF) regression-based models were trained and tested using the scikit-learn Python package (version 1.0.2). The radiogenomics discovery cohort was randomly divided into an internal training set (*n* = 139) and an internal independent test set (*n* = 35) according to 80 and 20%, and the validation cohort was used as the external test set (*n* = 72). In this work, optimization was performed for both feature pre-processing and model hyper-parameters. Five pre-processing operations were considered before training the model. The first operation was whether to normalize the image features using the Z-score method. The second one was whether to perform oversampling to remove the imbalance of the sample distribution. The specific process was performed by discrete the training set samples into 10 bins according to the enrichment scores of the target pathway and then balancing the number of samples in each interval by oversampling. The third operation was whether to construct the final training model using only the 15 most important image features determined by the 5-fold cross-validation training process. The fourth operation was whether to set weights on the samples based on the enrichment scores of the target pathway to balance the sample inhomogeneity. The final operation was whether to optimize the RF hyper-parameters automatically or just use the default hyper-parameters. If the model should be optimized, five important hyper-parameters of RF, including the number of trees, node criterion, max depth, max samples, and max features were optimized using gird search with 5-fold cross-validation and negative mean absolute error. The details of the RF parameters to be optimized in this work were as follows: n_estimators: [30, 50, 100, 250, 500, 1000, 2000]; criterion: [“absolute_error”, “squared_error”, “poisson”]; max_depth: [2, 4, 6, 8, 10, none]; max_samples: [0.2, 0.4, 0.6, 0.8, none]; and max_features: [0.2, 0.4, 0.6, 0.8, 1.0]. Five-fold cross-validation (CV) was applied to avoid overfitting. The pre-processing and machine learning analysis was performed in Python 3.8.10 (Virginia, USA).

### 2.8. Statistical Analysis

Two-sided Pearson’s chi-squared test or Fisher’s exact test were used to determine whether there was a significant difference between the two cohorts. The quantitative association between radiomic features and the GSVA enrichment scores of the pathways was given by the Pearson correlation coefficient. *P*-values smaller than 0.05 were considered significant. Mean absolute error (MAE) was used to evaluate the prediction performance of RF prediction models, and the 95% confidence interval (CI) was used to evaluate the performance stability during the 5-fold CV. The statistical analysis was performed using R 4.0.1 (Murray Hill, NJ, USA).

## 3. Results

### 3.1. Broadly Significant Associations between Hallmark Pathways and DCE-MRI Radiomic Features in BC

We performed statistical analysis and the presentation of both the demographic information of the radiogenomic cohorts and the significantly associated imaging features or hallmark pathways based on GSEA, respectively. Table 1 details the clinical characteristics of the two radiogenomic cohorts. Except for the PAM50 molecular subtypes, the two datasets had no differences in other characteristics. For the GSEA-based association analysis, we observed 101 radiomic features, and 43 hallmark pathways were significantly associated with GSEA (*p*-value < 0.05) in the discovery cohort, and 138 radiomic features and 41 hallmark pathways in the validation cohort (Table 2). The number of all of the significant qualitative association pairs of the two cohorts were 805 and 1114 (accounting for 9.3 and 12.8% of all possible pathway-feature combinations), respectively. Although the absolute proportions of significant associations were low, more than 80% of hallmark pathways in both cohorts (86 and 82%, respectively) showed significant imaging feature correlations (Table 2), demonstrating the widespread impact of the transcriptional activity on the phenotypes of complex disease. In addition, the proportion of radiomic features significantly associated with the hallmark pathways to all of the imaging features was 58 and 79.3%, respectively (Table 2). For a hallmark pathway, an average of 18.7 and 27.2 radiomic features were associated with it, respectively. Conversely, for a radiomic feature, there were averagely 7.9 and 8.1 hallmark pathways associated with it.

### 3.2. Radiomic Features of Tumor Shape and Mid-To-Late Enhanced Stages Showed More Transcriptional Associations

**Figure 2:**
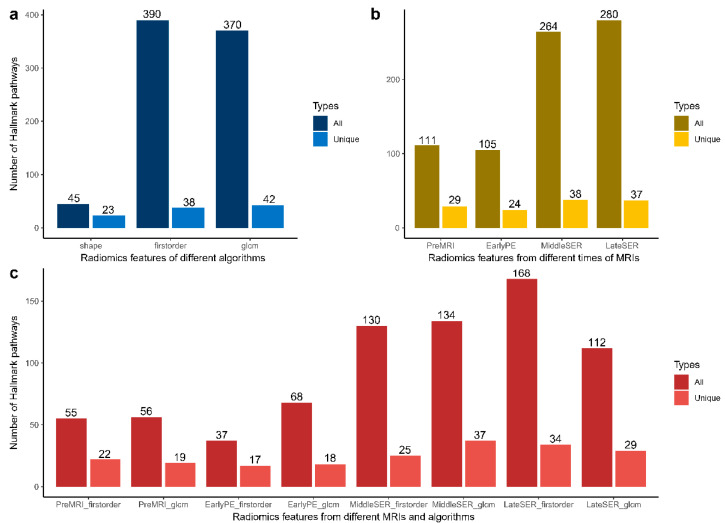
GSEA-based associations of different kinds of DCE-MRI features with hallmark pathways in the discovery cohort of BC radiogenomics. Imaging features were categorized into different classes according to three different definitions. The number of significant associations between different algorithm imaging features and hallmark pathways are displayed in (**a**), the number of significant associations between imaging features at different times during enhancement and hallmark pathways are shown in (**b**), and (**c**) presents the numbers of imaging features significantly related to hallmark pathways for different algorithms and at different times. As an imaging feature may be significantly associated with more than one hallmark pathway based on the GSEA, not only the absolute number of all significant associations was counted (type: All) but also the number of unique hallmark pathways was obtained by removing the duplicated pathways (type: Unique). Numbers represent the number of significant associations.

We further investigated whether imaging features related to hallmark pathway activity, identified by GSEA, differed significantly across the feature categories and reported these findings for the first time. Using the algorithm-based imaging feature category criteria (feature category I), 174 radiomic features were divided into shape, first-order, and GLCM features. The first-order and GLCM features contributed the most significant correlations, but the shape features had the highest ratio of transcriptional associations (Figure 2a, Figure S2a, and Table 2). We then compared the radiomic features extracted from MR images at different times (feature category II) and found that features from the SER images, at the time of late contrast enhancement, showed relatively high transcriptional associations in both cohorts (Figure 2b, Figure S2b, and Table 2). This temporal preference persisted when considering the features of different algorithms and images (feature category III), except for the validation cohort where PE images from early enhancement had more transcriptional associations (Figure 2c and Figure S2c). Furthermore, due to the possible one-to-multiple relationships, the number of unique radiomic-associated hallmark pathways of each type of DCE-MRI feature was calculated (Figure 2, Figure S2, and Table 3). The two-sided chi-squared test showed that there was no significant difference in the transcriptional association between different categories of imaging features in the two cohorts.

### 3.3. The Significant Radiomic-Associated Hallmark Pathways Demonstrated Molecular Function Preferences

**Figure 3:**
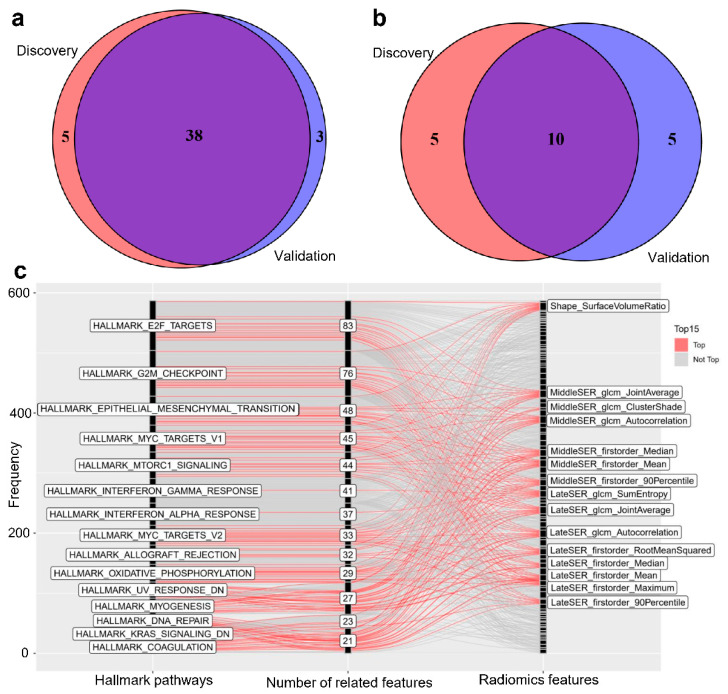
Hallmark pathways with significant imaging correlation were highly overlapping in both cohorts. The distribution of the pathways related to imaging features in the discovery and validation cohorts, shown in (**a**,**b**), presents the overlap of the top 15 pathways in the discovery dataset and the top 15 pathways in the validation dataset, as ordered by the number of significant associations. The details of the top 15 hallmark pathways and the top 15 DCE-MRI features of the discovery cohort are presented in (**c**), and the numbers on the middle axis represent the number of significant associations.

On the other hand, we attempted to identify qualitative GSEA-based imaging–transcriptomic associations that were stable across different datasets and uncover whether there is an overlap in the biological function between the hallmark pathways that have significant correlation with imaging features. The results from the two independent radiogenomic cohorts demonstrated a significant and stable relationship between the hallmark pathways and radiomic features (Figure 3 and Figure S3). The intersection of the number of pathways with the radiomic–transcriptomic association in the two cohorts was 38 (Figure 3a), and the intersection of the DCE-MRI radiomic features was 81 (Figure S3b). We first ranked the hallmark pathways by the number of significant associations with the radiomic features and selected the top 15 pathways in both radiogenomic cohorts. Ten hallmark pathways, including “E2F TARGETS”, “G2M CHECKPOINT”, “MYC TARGETS V1”, “MTORC1 SIGNALING”, “KRAS SIGNALING DN”, “EPITHELIAL MESENCHYMAL TRANSITION”, “MYOGENESIS”, “INTERFERON GAMMARESPONSE”, “INTERFERON ALPHA RESPONSE”, and “ALLOGRAFT REJECTION”, showed broad and stable imaging phenotype associations in both radiogenomic cohorts (Figure 3b–c and Figure S3a). For example, the pathway “E2F TARGETS” was remarkedly related to 83, and 84 DCE-MRI features in the discovery and validation cohorts, ranking first and third, respectively (Figure 3c and Figure S3a). Although imaging features significantly associated with pathway activity also overlap remarkably in both datasets, there was a notable difference in the ranking of the transcriptional associations of the DCE-MRI radiomic features in the two cohorts (Figure S2b–c).The first-order and GLCM features extracted from the middle and late SER images exhibited a high proportion of transcriptional correlations in the discovery cohort, yet in the validation cohort, the features of early PR images and tumor shape ranked high (Figure 3c and Figure S3a). This may be explained by the high system bias of the MRI data in the validation cohort.

In order to further explore the heterogeneity of imaging-related transcriptional activities, the 10 remarkable pathways were grouped into 4 categories based on their molecular functions. Cell cycle-related pathways included “E2F TARGETS” and “G2M CHECKPOINT”. Proliferation-related pathways consisted of “MYC TARGETS V1”, “MTORC1 SIGNALING”, and “KRAS SIGNALING DN”. Extracellular matrix (ECM)-related pathways included “EPITHELIAL MESENCHYMAL TRANSITION” and “MYOGENESIS”. Additionally, “INTERFERON GAMMA RESPONSE”, “INTERFERON ALPHA RESPONSE”, and “ALLOGRAFT REJECTION” attributed to the immune-related pathways. The four major categories were further analyzed using GSVA and demonstrated in our subsequent results.

### 3.4. The Association Heterogeneity between the Four Categories of Hallmark Pathways and Radiomic Features

**Figure 4:**
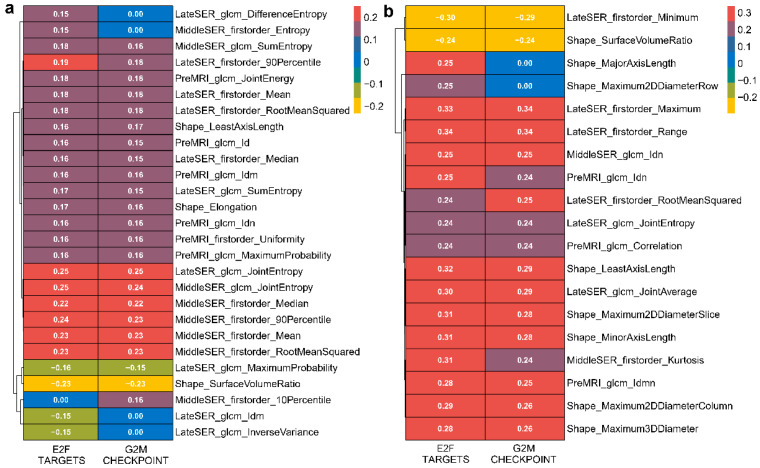
Quantitative associations of GSVA enrichment scores of two cell cycle-related hallmark pathways with imaging features. The significant Pearson correlation coefficients of the enrichment scores of both “E2F TARGETS” and “G2M CHECKPOINT” pathways with the imaging features for the discovery and validation cohorts are detailed in (**a**,**b**), respectively. Non-zero values in the cells represent significant correlation coefficients, and zero represents non-significant correlation coefficients.

**Figure 5:**
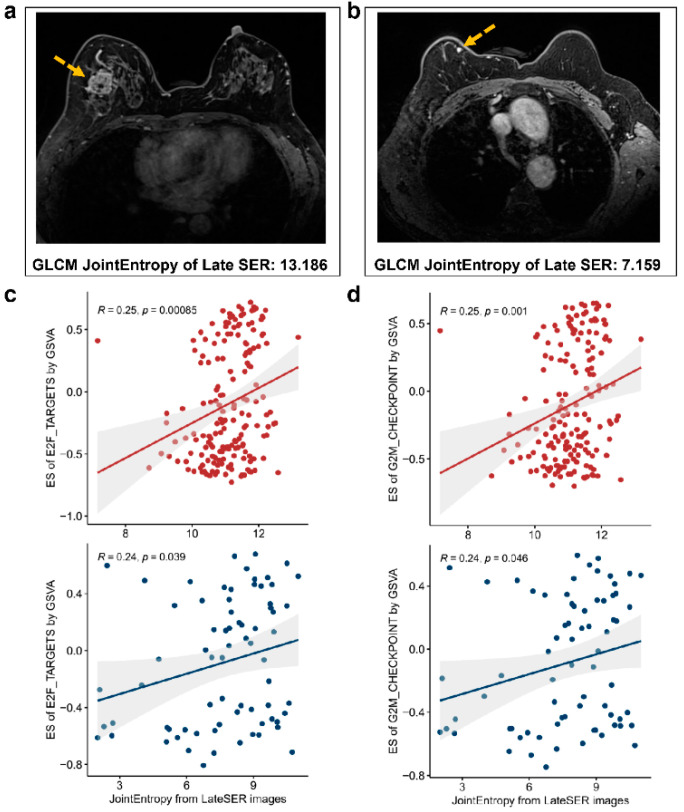
Clinical imaging differences were associated with the expression heterogeneity of hallmark pathways. The MR image of a patient with a high joint entropy value of GLCM for late SER image is illustrated in (**a**), and in (**b**) the MR image of a patient with a low value of this feature is displayed (The yellow arrows point to the tumors). (**c**,**d**) show the scatter plots of the values of joint entropy in breast cancer patients in the discovery set and validation set versus the enrichment scores of the “E2F TARGETS” and “G2M CHECKPOINT” pathways, respectively.

To quantify the radiomic–transcriptomic correlation, we calculated and reported the Pearson correlation coefficient of each hallmark pathway enrichment score obtained using GSVA with each DCE-MR imaging feature value. Firstly, many significant positive Pearson correlations between cell cycle-related pathways and imaging features were observed in both the discovery and validation cohorts (Figure 4). It was noteworthy that the GLCM feature JointEntropy from the late SER map had the highest Pearson correlation coefficient values of 0.25 and 0.25 in the discovery cohort and 0.24 and 0.24 in the validation cohort with the expression variation scores of cell cycle-related pathways “E2F TARGETS” and “G2M CHECKPOINT”, respectively (Figure 4a,b). We further illustrated the differences in imaging between patients with high and low late SER JointEntropy (Figure 5a,b) and revealed that patients with higher late SER JointEntropy showed significantly higher enrichment scores in the cell cycle-related pathways, such as “E2F TARGETS” and “G2M CHECKPOINT” (*p*-value < 0.05, Figure 5c,d). Our results demonstrate that the relationship between the late SER JointEntropy and the two hallmark pathways exhibited a high degree of consistency and robustness in both datasets. In addition, the tumor-shape feature SurfaceVolumeRatio exhibited the greatest negative relationships with the GSVA scores of “E2F TARGETS” and “G2M CHECKPOINT”, as the Pearson correlation coefficients (*p*-value < 0.05) of −0.23 and −0.23 were found for the discovery cohort and −0.24 and −0.24 for the validation cohort, respectively (Figure 4a,b).

Three proliferation-related pathways were found related to more tumor-size features than other hallmark pathways (Figure S4a and Figure S5a). Two tumor-size features, including LeastAxisLength and MinorAxisLength, both indicating the minimum size of the tumor, were significantly positively related to the “MTORC1 SIGNALING” pathway (*p*-value < 0.05). Interestingly, many significantly negative correlations between the two ECM-related pathways (“EPITHELIAL MESENCHYMAL TRANSITION” and “MYOGENESIS”) and radiomic features were revealed (Figure S4b and Figure S5b). The GLCM feature Idn, from the pre-contrast MRI, showed the most significant negative correlation with both pathways, with the r values of −0.27 and −0.3, respectively (*p*-value < 0.05, Figure S4b). In addition, most of these negatively correlated imaging features, such as the first-order feature Mean, Median, 90Percentile, and 10Percentile from the late SER map, were associated with tumor signal enhancement (Figures S4b and S5b). For the immune-related pathways in the discovery cohort, most of the pathway-related features were extracted from both the middle and late SER maps (Figure S4c); however, the significantly correlated features in the validation dataset were mainly from the early PE images (Figure S5c). Despite some discordant results, overall, the significant correlations between the DCE-MRI features and GSVA scores of the hallmark pathways were identified and validated for the first time.

### 3.5. Non-Invasive Prediction of the Enrichment Scores of GSVA of Hallmark Pathways

**Figure 6:**
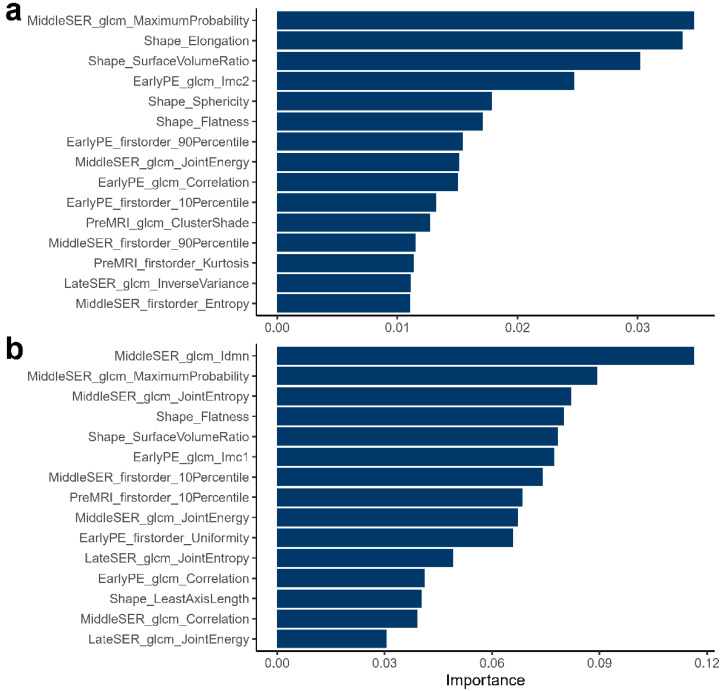
The top 15 important radiomic features for the random forest prediction models of representative hallmark pathways. The top 15 important DCE-MRI features of the G2M checkpoint prediction model and the mTORC1 signaling prediction model are detailed in (**a**,**b**), respectively.

To extend the application scenario of radiomics, for the first time, in this work, we attempted to use imaging features to build machine-learning models for predicting the transcriptomic characteristics at the level of pathways in BC, namely, predicting the enrichment scores (ESs) of the GSVA in the pathways. The RF prediction models were established and validated for four representative hallmark pathways. The model was trained in the internal training set (*n* = 139) and tested in the internal test set (*n* = 35) and external multi-center test set (*n* = 72). The training and testing MAEs of the RF regressors are reported in Table 4. The best-performing constructs of the RF models are detailed in Table S1. Compared to the other models, the RF model for the mTORC1 signaling pathway had the lowest MAEs, with 27.42% in the five-fold CV training set, and 27.29 and 28.61% in the internal and external test sets, respectively. Notably, the RF regressors for cell cycle-, proliferation-, and ECM-related pathways showed similar performances in the internal and external datasets (Table 4). However, the prediction model for the immune-related pathway (INTERFERON GAMMA RESPONSE) performed significantly worse in the external test set (40.37%) than in the internal test set (36.82%). This was consistent with the results of the GSEA-based analysis in that the association of the immune-related pathways with imaging features was more unstable. The top 15 important radiomic features for the prediction models of “G2M CHECKPOINT”, “MTORC1 signaling”, “Epithelial mesenchymal transition”, and “INTERFERON GAMMA RESPONSE” are displayed in Figure 6a–b and Figure S6a–b, respectively. The important imaging features with predictive ability demonstrated remarkable pathway correlations. For example, the GLCM, featuring MaximumProbability from the middle SER image, was the most important feature in the RF regressor of “G2M CHECKPOINT” (Figure 6a) and significantly negatively associated with the ES of GSVA (Figure 4a). Tumor-shape characteristics such as SurfaceVolumeRatio and Flatness were important features in all of the prediction models (Figure 6a,b and Figure S6a,b), suggesting that tumor-shape features, as simple features that can be readily available in clinical practice, may have more application.

## 4. Discussion

In the present work, we investigated, for the first time, the associations of DCE-MRI radiomic features with hallmark pathway expression activities in BC based on two independent datasets. Our results indicate that more than 80% of hallmark pathways significantly correlated with imaging features in both cohorts, and at least half of the imaging features showed the relevance of transcriptional activity. Tumor-shape features and imaging features extracted from the middle and late images in the contrast enhancement exhibited broader correlations with hallmark pathway activities. The hallmark pathways associated with radiomics showed a similar preference in biological functions. Cell cycle-, proliferation-, ECM-, and immune system-related hallmark pathways have broader and more remarkable imaging associations. Moreover, we made the first attempt to develop non-invasive RF regression models of the enrichment scores of hallmark pathways in BC patients based on radiomic features, which may be useful for personalized medicine for BC.

Radiomics is an emerging field in translational medicine that is gaining increasing interest in the practice of precision medicine for a variety of cancers, such as breast cancer and prostate cancer, because of its ability to provide a promising approach to non-invasive predictions of the characterization of key gene mutation, molecular activity, progression, treatment response, and prognosis of disease [36,37,38,39]. The associations of DCE-MRI imaging features with transcriptomic activities in BC have been reported in many studies. For example, the enhancement pattern in DCE-MRI suggested the deregulation of the mTOR pathway [40], and the enhancing rim fraction score can indicate the early metastasis of BC and expression of some important long non-coding RNAs [41]. Compared to the pre-contrast and early post-contrast images, we found that radiomic features from the middle and late post-contrast images were more transcriptomic-related in the discovery cohort (Figure 2), which might be because, in the mid or late stage of dynamic contrast enhancement, the contrast agent was sufficiently absorbed and metabolized in the tumor tissue and its microenvironment to accurately reflect the imaging features of the tumor. However, features from the early post-contrast images displayed more transcriptional correlations in the validation cohort (Figure S2). This might be caused by the inconsistent acquisition time points during DCE-MRI, with the images from the local database using the newest MRI protocols, whose first post-contrast acquisition time was around 1 min, whereas the validation dataset from the public database used the old imaging protocol, whose first post-contrast adoption time point was much later. In addition, the tumor-shape features were found to be most closely connected to the hallmark transcriptional activity in both cohorts (Table 2). This result was reasonable because shape features were the most basic tumor characteristics and were affected and regulated by various transcriptional activities [22]. Although the two cohorts originated from different medical centers, the association of the imaging features with the transcriptional activity did not differ significantly between the two cohorts when the imaging features were analyzed by different types. This illustrated the robustness of the associations between imaging features and hallmark expression activities (Table 3).

Previous radiogenomic studies of BC showed that the expression activities of some genes or the Kyoto Encyclopedia of Genes and Genomes (KEGG) pathways with specific functions were associated with imaging features [20,22]. In particular, KEGG pathways such as cell cycle, ECM–receptor interaction, and primary immunodeficiency have been frequently reported to be correlated with DCE-MRI imaging features [21,22]. In this work, we used hallmark gene sets instead of KEGG pathways to investigate the radiomic associations because the hallmark pathways are noise-removed and more representative. We found that most of the hallmark pathways were related to the DCE-MRI imaging features (Table 2) and that the top-ranked pathways in both cohorts had high overlap (Figure 3). By looking at the detailed biological functions involved in these notable pathways, we further found that they can be classified into four major categories, including cell cycle-, proliferation-, ECM-, and immune-related pathways. In our work, the E2F target pathways exhibited the broadest imaging correlation, particularly a significant negative correlation with the surface-to-volume ratio and significant positive correlations with the joint entropy of the middle and late SER images (Figure 3 and Figure 4). The E2F transcription factors regulate a wide range of genes that are essential for DNA replication and cell-cycle progression and are closely involved in the progression and metastasis of BC [42,43]. Recent research also showed that the E2F targets pathway score is a potential biomarker of a neoadjuvant therapy response in BC patients [44]. As a recently revisited hallmark of cancer development, MYC transcription factors regulate almost every aspect of cellular metabolism and are dysregulated in most types of cancers [45]. MYC can also promote tumor proliferation in BC, and it may be a potential therapeutic target of triple-negative BC patients [46]. The mTORC1 signaling pathway is a widely studied pathway with important roles in cellular protein synthesis and autophagy and is closely associated with BC metastasis and response to targeted therapies [47,48]. In addition, as an essential component of the tumor microenvironment, the ECM likewise plays an important role in mediating the progression and metastasis of BC, which may influence the process of enhanced imaging [49,50,51]. Playing a dual role in tumor suppression and pro-tumorigenesis in human cancers, interferon gamma is engaged in complex tumor immune regulatory mechanisms and is associated with the immune microenvironment and prognosis of BC [52,53,54]. Our results suggest that these hallmark gene sets, which are intimately involved in the processes of BC development, progression, metastasis, and treatment response, have clear and robust relationships to the radiomic features of DCE-MRI.

The non-invasive prediction of key molecular characteristics or clinical parameters of cancer is the core task of radiomics towards clinical application. Many efforts have been made to predict the receptor status, clinical or molecular subtypes, and other important disease features in BC patients [24,55,56,57]. However, few studies have focused on the non-invasive prediction of heterogeneity at the pathway level, and the vast majority of these studies were binary classification or multi-classification tasks, which are still insufficient for individualized medicine where higher precision is required. In this work, we made a preliminary attempt using DCE-MRI radiomic features to predict the enrichment scores of hallmark pathways for BC individuals. Although the random forest regression models showed poor prediction performance in both the internal and external test sets overall, the model of the mTORC1 signaling pathway performed reasonably in predicting the enrichment scores, with MAEs of 27.29 and 28.61% for the internal and external test sets, respectively (Table 4). Compared to the other three pathways, the prediction model for the interferon gamma response pathway performed significantly worse in the external dataset than in the internal dataset, probably due to the high heterogeneity of the individual immune system. Moreover, the important imaging features with predictive ability selected by RF were found to be highly overlapping with the significantly associated features obtained based on GSEA and GSVA (Figure 6 and Figure S6), which may improve the interpretability of the machine-learning models. It was also to be noted that the models did not perform very well during the training process of the five-fold CV (Table 4). The results might indicate that the imaging features we used in this work were not yet representative and that the accurate prediction of the expression heterogeneity for a specific pathway was difficult.

There were some limitations to this work. Firstly, the external cohort had a small sample size and was collected from multiple medical centers. Although we performed the required data normalization preprocessing and validated some radiomics–transcriptomics correlations in the cohort, a more refined image preprocessing and normalization approach is needed to better address data bias in a larger dataset. Secondly, we simply demonstrated and validated the association of DCE-MRI radiomic features with hallmark pathway activities in BC and analyzed the heterogeneity of this association. How to more effectively integrate these findings into clinical practice needs to be further explored in the future. Finally, the performance of the pathway enrichment score prediction models based on DCE-MRI radiomic features was still low, and the models can be further optimized by collecting larger study cohorts and using more radiomic features or deep features based on deep learning algorithms in future works.

## 5. Conclusions

In this work, the associations of DCE-MRI radiomic features with hallmark pathway expression activities were investigated and validated in two BC radiogenomic cohorts. There was a broad and significant relationship between imaging features and hallmark expression heterogeneity. The imaging features of tumor shape and mid-to-late post-contrast stages showed more transcriptional connections. Ten hallmark gene sets of cell cycle-, proliferation-, ECM-, and immune-related pathways exhibited more significant and tighter correlations with DCE-MRI radiomic features. We further developed prediction models for the expression enrichment scores of hallmark pathways based on radiomic features to expand the clinical application scenario of radiogenomics. Generally, our findings suggest that there was a significant correlation between the radiomic features and BC transcriptional activity and that this correlation can be validated and predicted in an external dataset. In addition, we believe that as more data are generated and combined with artificial intelligence technologies, such as deep learning, in the future, radiomics will certainly play an essential role in the clinical management of diseases throughout their stages, including early diagnosis, the monitoring of key molecular features, the selection of treatment strategies, and the evaluation of prognostic risk.

## Figures and Tables

**Table 1:** The clinical and molecular characteristics of BC tumors in the radiogenomic discovery and validation cohorts.

Characteristics	Discovery Cohort (*n* = 174)	Validation Cohort (*n* = 72)	*p*-Value
**Age, mean (SD)**	≤50y: 95/>50y: 79; 49.78y (9.99)	≤50y: 30/>50y: 42; 53.96y (11.75)	0.088 ^a^
**IHC receptors** ER status PR status HER2 status Ki67 status	P:127/N:47 P:111/N:63 P:36/N:138 high:136/low:38	P:61/N:11 P:55 N:17 P:14 N:37/NA:21 NA	0.071 ^a^ 0.077 ^a^ 0.407 ^a^ NA
**IHC-based subtype** Luminal-A Luminal-B HER2-positive Triple-negative	28 101 15 30	NA NA NA NA	NA NA NA NA
**PAM50 subtype** Luminal-A Luminal-B HER2-enriched Basal-like Normal-like	49 43 29 43 10	44 9 5 10 4	<0.001 ^b^
**Pathological stage** Stage I Stage II Stage III	55 94 25	17 47 8	0.267 ^a^

Note: unless otherwise indicated, data are the number of patients or P-value of the statistical test. ^a^ *p*-value for the two-sided Pearson’s chi-squared test, ^b^ *p*-value for the two-sided Fisher’s exact test. *P* for positive and N for negative. NA: not available.

**Table 2:** Percentages of hallmark pathways or imaging features with radiomic–transcriptomic associations. The proportion of pathways with significant imaging correlation with all 50 hallmark pathways, and the proportions of DCE-MRI features with significant transcriptomic association with all imaging features of that type were calculated and displayed for both cohorts.

Pathways or Features	Discovery Cohort	Validation Cohort
Unique associated hallmark pathways	43 (86.0%)	41 (82.0%)
Unique associated DCE-MRI radiomic features Shape features First-order features GLCM features Pre-MR images Early PE images Middle SER images Late SER images	101 (58.0%) 9 (64.3%) 46 (63.9%) 46 (52.3%) 18 (11.3%) 21 (13.1%) 25 (15.6%) 28 (16.3%)	138 (79.3%) 14 (100.0%) 56 (77.8%) 68 (77.3%) 33 (18.3%) 34 (21.3%) 26 (17.5%) 31 (19.4%)

Note: unless otherwise indicated, data are the number of hallmark pathways or DCE-MRI features which showed a statistically significant radiomic–transcriptomic association. The percentages of significant pathways or features to the total number of pathways or features of the respective type to which they belong are in parentheses.

**Table 3:** The number of radiomic-associated hallmark pathways of each type of DCE-MRI feature. The number of significant imaging-correlated hallmark pathways was counted for each class of imaging features in both cohorts, and whether the radiomic–transcriptomic associations were significantly different in the two independent datasets is shown.

Characteristics	Discovery Cohort	Validation Cohort	*p*-Value
**Feature category I** Shape features First-order features GLCM features	23 38 42	27 37 39	0.801
**Feature category II** Pre-MR images Early PE images Middle SER images Late SER images	29 24 38 37	29 36 31 32	0.324
**Feature category III** First-order from pre-MR images GLCM from pre-MR images First-order from early PE images GLCM from early PE images First-order from middle SER images GLCM from middle SER images First-order from late SER images GLCM from late SER images	22 19 17 18 25 37 34 29	25 26 27 34 30 26 30 22	0.129

Note: unless otherwise indicated, data are the number of unique hallmark pathways or the *p*-value of the two-sided Pearson’s chi-squared test.

**Table 4:** The prediction performance of the GSVA enrichment scores of hallmark pathways based on radiomic features. The random forest prediction models of enrichment scores for four representative hallmark pathways were built in the internal training set and validated in the independent internal and external test sets. Five-fold cross-validation (CV) was performed to avoid overfitting, and MAE was used to evaluate model performance. The 95% CI was used to evaluate the performance stability during the 5-fold CV.

Hallmark Pathways	Training Set (5-Fold CV)	Internal Test Set (*n* = 35)	External Test Set (*n* = 72)
**G2M checkpoint** MAE	32.86% (6.5%, 59.6%)	37.43%	37.22%
**Epithelial mesenchymal transition**			
MAE	33.11% (31.2%, 35.1%)	33.04%	36.25%
**MTORC1 signaling** MAE	27.42% (23.3%, 31.5%)	27.29%	28.61%
**Interferon gamma response** MAE	35.12% (8.4%, 62.2%)	36.82%	40.37%

Note: unless otherwise indicated, data are the mean absolute errors (MAEs) for each random forest regression prediction model. Values in parentheses are 95% confidence intervals (CIs) for the MAEs of the 5-fold CV.

## Data Availability

The RNA-seq data of the radiogenomics discovery cohort are available at https://ngdc.cncb.ac.cn/bioproject/ (GSA-Human number: HRA001100, accessed on 1 September 2019). The gene expression and MRI data of the validation cohort are available at https://portal.gdc.cancer.gov/projects/TCGA-BRCA and https://wiki.cancerimagingarchive.net/display/Public/TCGA-BRCA (accessed on 1 January 2020). Further information and other data that support the findings of this study are available from the corresponding author upon reasonable request.

## Supplementary Materials

The following supporting information can be downloaded at: https://www.mdpi.com/article/10.3390/genes14010028/s1, Figure S1: The inclusion and exclusion criteria of BC patients for radiogenomic cohorts. Figure S2: GSEA-based associations of different kinds of DCE-MRI features with hallmark pathways in the validation cohort of BC radiogenomics. Figure S3: Imaging features significantly associated with pathway activity were consistent to some extent in both cohorts. Figure S4: Quantitative associations of GSVA enrichment scores of hallmark pathways with imaging features in the discovery cohort. Figure S5: Quantitative associations of GSVA enrichment scores of hallmark pathways with imaging features in the validation cohort. Figure S6: The top 15 important radiomic features for the random forest prediction models of hallmark pathways. Table S1:.The best-performed pre-processing configures of RF models.
