# Supplementary material for: Identifying Associations between DCE-MRI Radiomic Features and Expression Heterogeneity of Hallmark Pathways in Breast Cancer: A Multi-Center Radiogenomic Study
Source: Genes (Basel). 2022 Dec 22;14(1):28. doi: 10.3390/genes14010028 (PMC9858814; doi:10.3390/genes14010028)
Supplement: Supplementary file 1 [file genes-14-00028-s001.zip › genes-2043776-supplementary.pdf]

Supplementary Materials of

Identifying associations between DCE-MRI radiomics features and expression heterogeneity of Hallmark pathways in breast cancer: a multi-center radiogenomics study

Wenlong Ming, Yanhui Zhu, Fuyu Li, Yunfei Bai, Wanjun Gu, Yun Liu, Xiao Sun, Xiaoan Liu, Hongde Liu

*Corresponding email: liuhongde@seu.edu.cn

Figure S1. The inclusion and exclusion criteria of BC patients for radiogenomics cohorts.

Figure S2. GSEA-based associations of different kinds of DCE-MRI features with Hallmark pathways in the validation cohort of BC radiogenomics.

Figure S3. Imaging features significantly associated with pathway activity were consistent to some extent in both cohorts.

Figure S4. Quantitative associations of GSVA enrichment scores of Hallmark pathways with imaging features in the discovery cohort.

Figure S5. Quantitative associations of GSVA enrichment scores of Hallmark pathways with imaging features in the validation cohort.

Figure S6. The top 15 important radiomics features for the random forest prediction models of Hallmark pathways.

Table S1. The best-performed pre-processing configures of RF models.

Figures

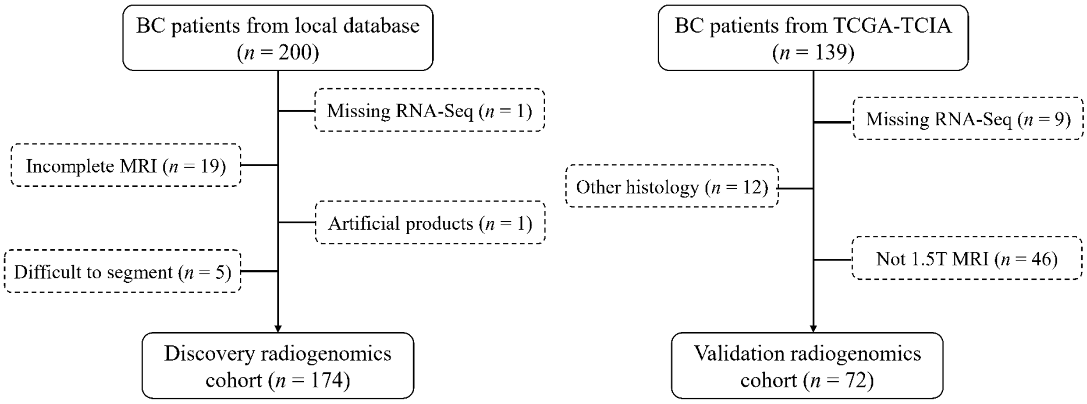

Figure S1. The inclusion and exclusion criteria of BC patients for radiogenomics cohorts. In this work, 246 BC patients from local and public databases were enrolled for radiogenomics study.

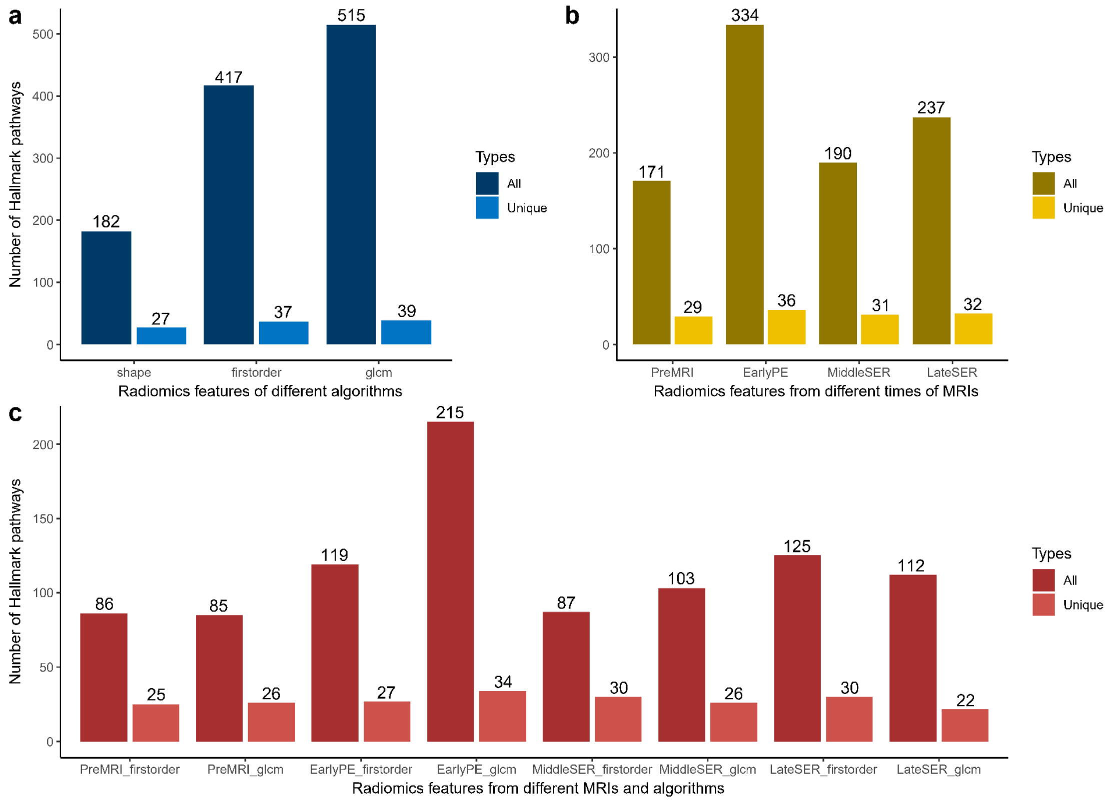

Figure S2. GSEA-based associations of different kinds of DCE-MRI features with Hallmark pathways in the validation cohort of BC radiogenomics. Imaging features were categorized into different classes according to three different definitions. The numbers of significant associations between different algorithm imaging features and Hallmark pathways were displayed in **a**, the numbers of significant associations between imaging features of different times during enhancement and Hallmark pathways were shown in **b**, and **c** presented the numbers of imaging features significantly related to Hallmark pathways for different algorithms and different times. Since an imaging feature may be significantly associated with more than one Hallmark pathway based on the GSEA, not only the absolute number of all significant associations was counted (type: All), but also the number of unique Hallmark pathways was obtained by removing the duplicated pathways (type: Unique). Numbers represented the number of significant associations.

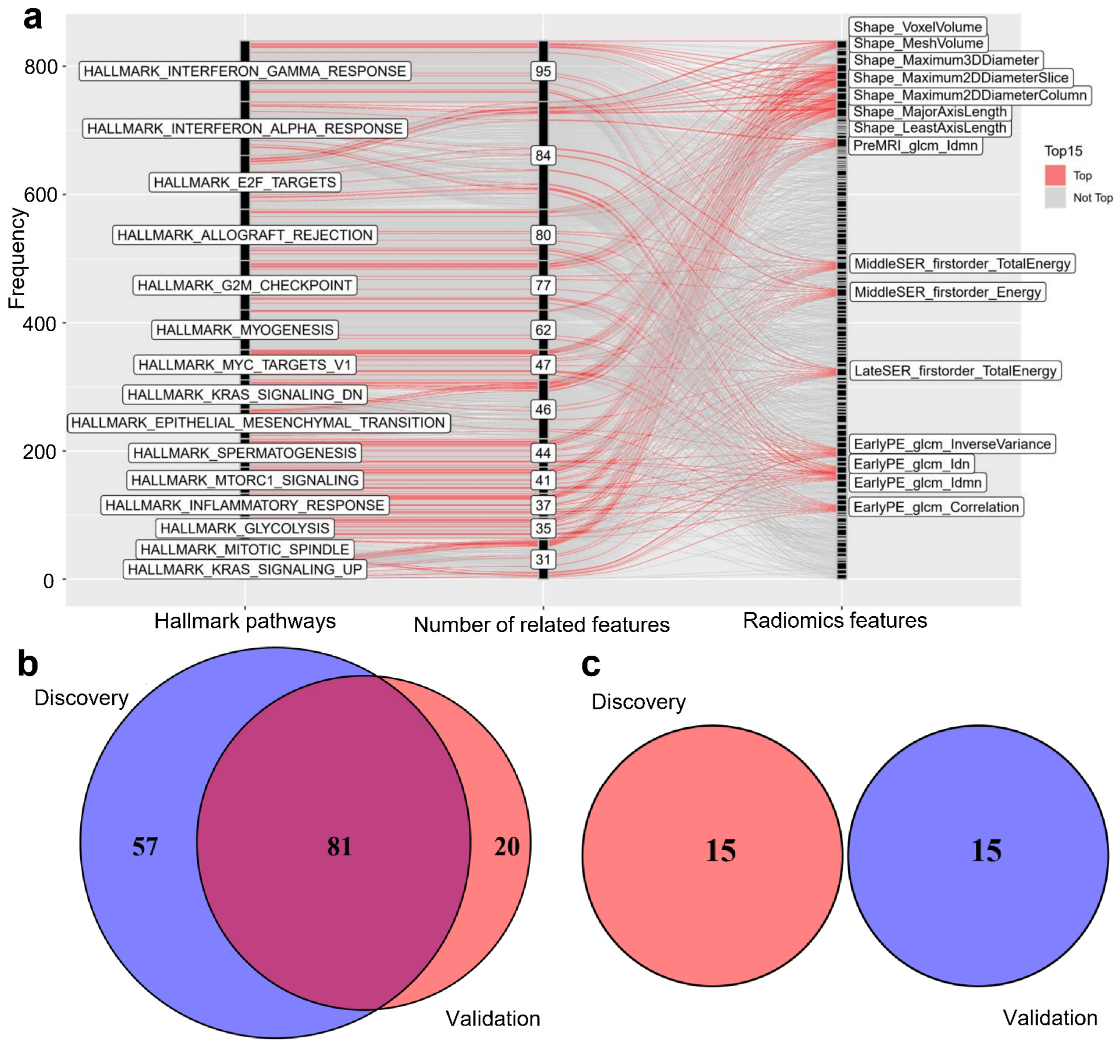

Figure S3. Imaging features significantly associated with pathway activity were consistent to some extent in both cohorts. The details of the top 15 Hallmark pathways and the top 15 DCE-MRI features of the validation cohort were presented in **a**, and the numbers on the middle axis represented the number of significant associations. The distribution of the imaging features related to pathways in the discovery and validation cohorts was shown in **b**, and **c** presented the overlap of the top 15 features in the discovery dataset and the top 15 features in the validation dataset, as ordered by the number of significant associations.

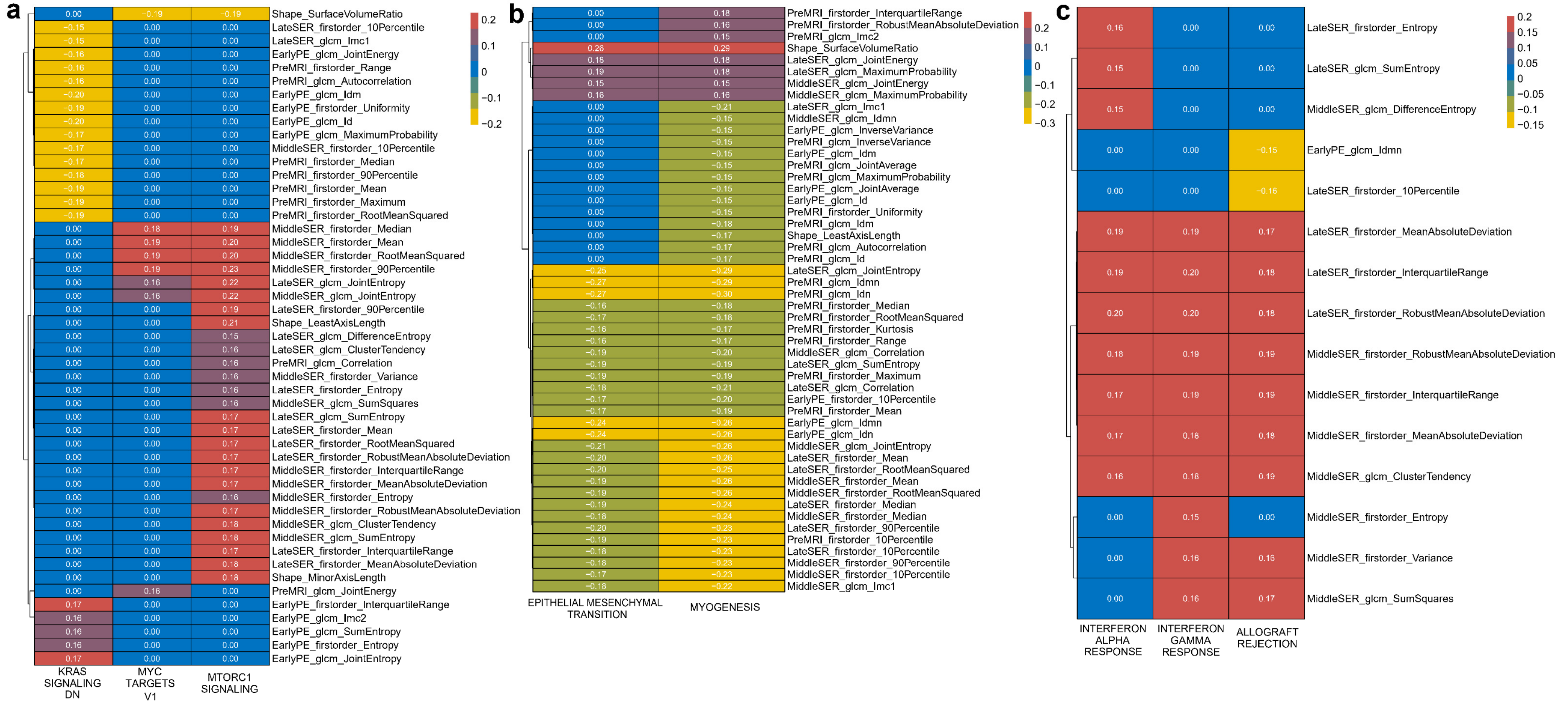

Figure S4. Quantitative associations of GSVA enrichment scores of Hallmark pathways with imaging features in the discovery cohort.

The significant Pearson correlation coefficients of the enrichment scores of proliferation-related, ECM-related, and immune-related pathways with

the imaging features were detailed in **a**, **b**, and **c**, respectively. Non-zero values in the cells represent significant correlation coefficients and zero represents non-significant.

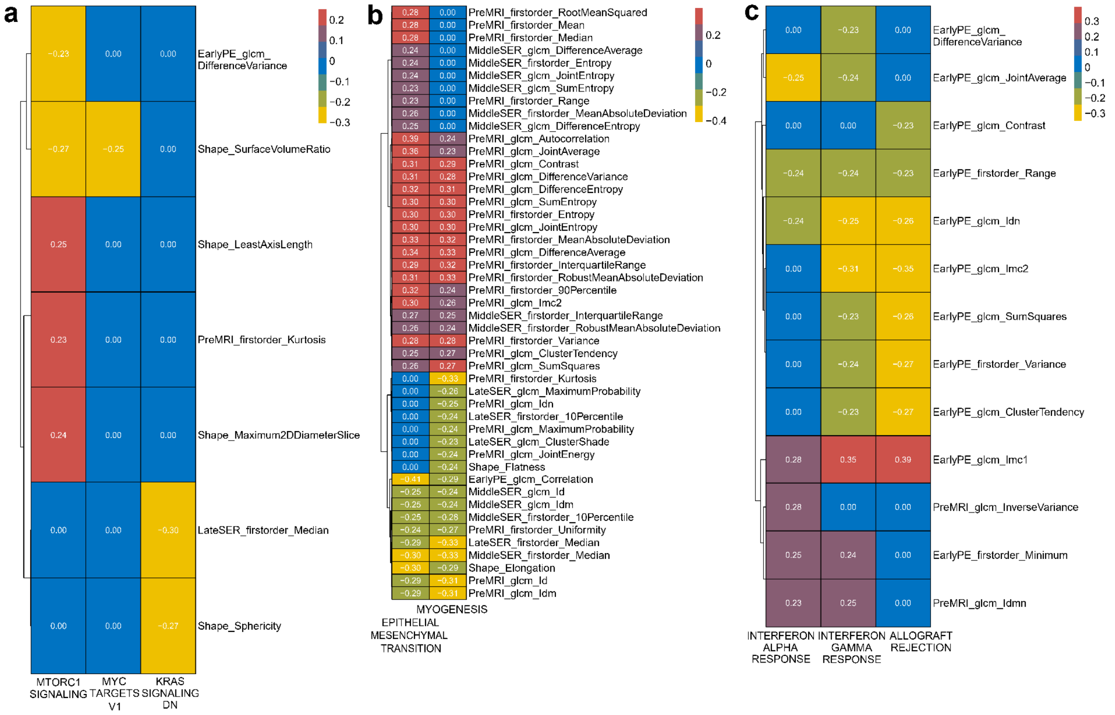

Figure S5. Quantitative associations of GSVA enrichment scores of Hallmark pathways with imaging features in the validation cohort. The significant Pearson correlation coefficients of the enrichment scores of proliferation-related, ECM-related, and immune-related pathways with the imaging features were detailed in **a**, **b**, and **c**, respectively. Non-zero values in the cells represent significant correlation coefficients and zero represents non-significant

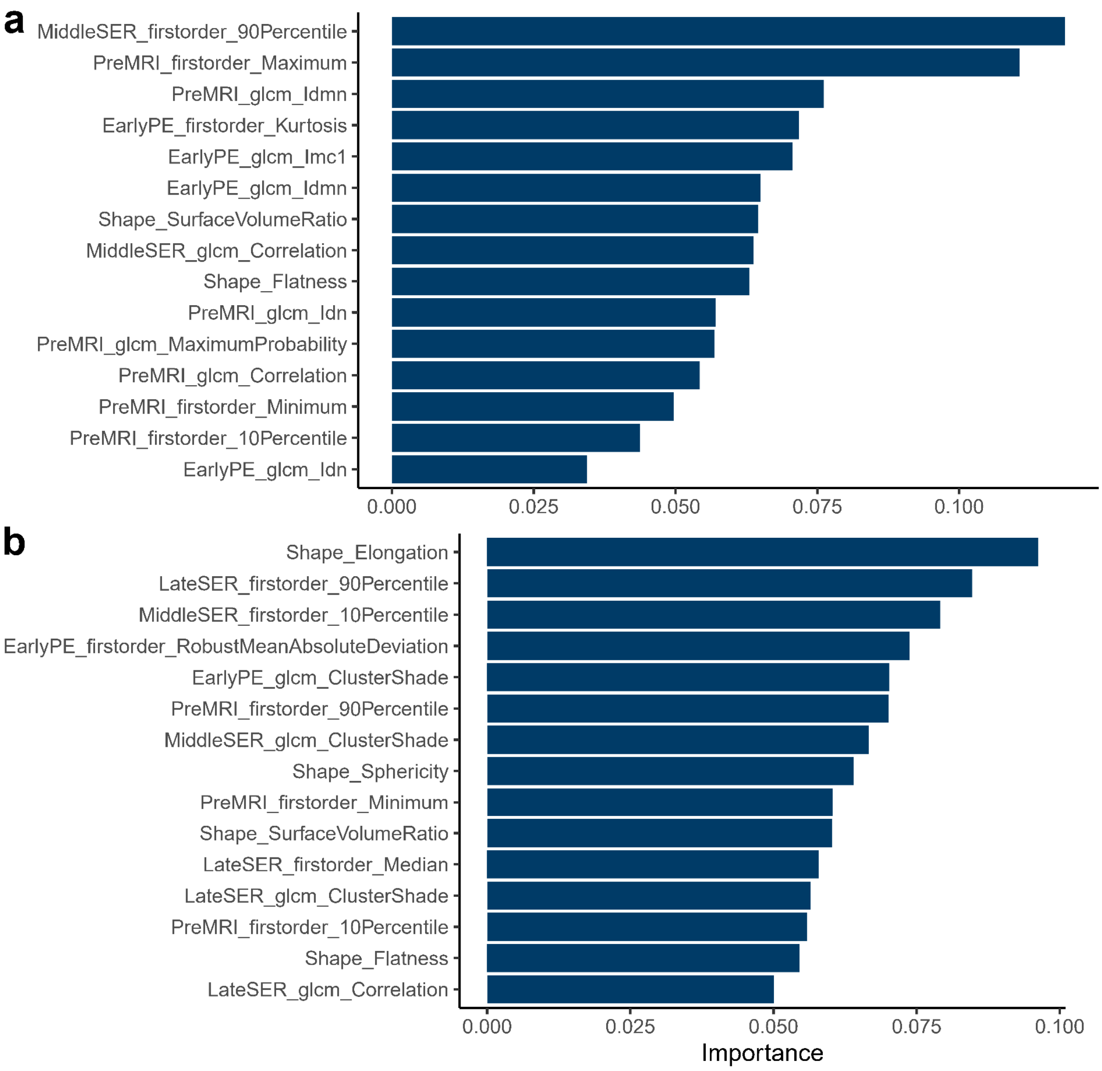

Figure S6. The top 15 important radiomics features for the random forest prediction models of Hallmark pathways. The top 15 important DCE-MRI features of the epithelial mesenchymal transition prediction model and the interferon gamma response prediction model were detailed in **a** and **b**, respectively.

Tables

Table S1. **The best-performed pre-processing configures of RF models.** The detailed feature pre-processing parameters for each optimal model in the 5-fold CV training set.

**Models**	**Normalize**	**Oversample**	**Features**	**Optimizer**	**Sample Weighting**
**G2M checkpoint**	Yes	Yes	All features	Yes	No
**Epithelial mesenchymal transition**	No	No	Top15 features	Yes	Yes
**MTORC1 signaling**	No	No	Top15 features	Yes	No
**Interferon gamma response**	Yes	Yes	Top15 features	Yes	No

Note: ‘Yes’ means perform the pre-processing operation, ‘No’ means do not perform it.
